# Neurological complications in HIV patients – a case of PML-IRIS

**DOI:** 10.1186/1471-2334-14-S7-P93

**Published:** 2014-10-15

**Authors:** Smaranda Gliga, Mathilde Devaux, Robert Carlier, Christian Perronne, Benjamin Davido

**Affiliations:** 1 National Institute for Infectious Diseases "Prof. Dr. Matei Balş", Bucharest, Romania; 2 Raymond Poincaré Teaching Hospital, AP-HP, Garches, France

## Background

Progressive multifocal leukoencephalopathy (PML) is a rare but frequently fatal demyelinating disease caused by the JC polyomavirus (JCV). It has been traditionally associated to severe immunosuppression and described mainly in HIV patients with a low CD4 count. On neuroimaging, PML is classically seen as diffuse, often multifocal, white matter lesions involving the U-fibers without associated contrast enhancement, vasogenic edema or mass effect. If untreated, PML is usually fatal within 1 year. Treatment with highly active antiretroviral therapy (HAART) may prolong survival. Nevertheless, in the recent decade, the availability of HAART can also be responsible for some PML cases attributed to the reconstitution of the immune system known as PML-IRIS.

## Case report

We report the case of a 32 year-old man, originating from Ghana, who, following investigation of chronic renal insufficiency was diagnosed with HIV-HBV co-infection. The immuno-virological status at baseline showed a high HIV1 viral load (247,000 copies/mL) and low CD4 cell count=11/cmm. In adjunction to *Pneumocystis* prophylaxis with pentacarinat aerosols, HAART was initiated with lamivudine/abacavir + darunavir/r BID. Two weeks after initiation of HAART he presented with progressive dysphagia, fever and mild left facial palsy of central type. His blood tests showed a decrease in viral load >2 log and CD4 cell count=79/cmm. The neurological deficit rapidly progressed to the left brachial and crural regions. There was a high suspicion of toxoplasmosis. However, neuroimaging (MRI) revealed diffuse plus white matter lesions involving the U-fibers suggestive of PML but atypical because of contrast enhancement and a mass effect on the left ventricular. Hence we hypothesized the patient could be suffering of an IRIS. The lumbar puncture parameters were: cells 7/cmm, proteins 0.31 g/L, glucose 3.1 mmol/L and a positive PCR for JCV (360 copies/mL). The diagnosis of PML motivated the addition of maraviroc and raltegravir BID to the therapy. The outcome was favorable in the absence of corticosteroid therapy, with slow but complete regression of the brachial and crural motor deficit after 1 month. Dysphagia (bilateral paralysis of the IX^th^ and XII^th^ cranial nerves) persisted for several months, requiring temporary installation of a gastrostomy.

## Conclusion

PML continues to be one of the most fatal opportunistic infections in HIV patients. The presence of peripheral low contrast enhancement on neuroimaging, a low JCV viral load in the CSF and IRIS unmasking PML are considered good prognostic factors. Corticosteroid use in PML-IRIS remains controversial.

## Consent

Written informed consent was obtained from the patient for publication of this Case report and any accompanying images. A copy of the written consent is available for review by the Editor of this journal.

